# Warfarin Accelerates Aortic Calcification by Upregulating Senescence-Associated Secretory Phenotype Maker Expression

**DOI:** 10.1155/2020/2043762

**Published:** 2020-10-22

**Authors:** Ningle Wei, Liuyi Lu, Huanji Zhang, Ming Gao, Sounak Ghosh, Zhaoyu Liu, Junhua Qi, Jingfeng Wang, Jie Chen, Hui Huang

**Affiliations:** ^1^Guangdong Provincial Key Laboratory of Malignant Tumor Epigenetics and Gene Regulation, Department of Cardiology, Sun Yat-sen Memorial Hospital, Sun Yat-sen University, Guangzhou 510120, China; ^2^Department of Cardiology, The Eighth Affiliated Hospital, Sun Yat-sen University, Shenzhen 518033, China; ^3^Department of Radiation, Sun Yat-sen Memorial Hospital of Sun Yat-sen University, Guangzhou 510120, China; ^4^Department of Pharmacy, Guangzhou Women and Children' s Medical Center, Guangzhou 510120, China; ^5^Department of Radiotherapy, Sun Yat-sen Memorial Hospital of Sun Yat-sen University, Guangzhou 510120, China

## Abstract

Warfarin, a vitamin K antagonist (VKA), is known to promote arterial calcification (AC). In the present study, we conducted a case-cohort study within the Multi-Ethnic Study of Atherosclerosis (MESA); 6655 participants were included. From MESA data, we found that AC was related to both age and vitamin K; furthermore, the score of AC increased with SASP marker including interlukin-6 (IL-6) and tumor necrosis factor alpha (TNF-*α*) rising. Next, a total of 79 warfarin users in our center developed significantly more calcified coronary plaques as compared to non-VKA users. We investigated the role of warfarin in phosphate-induced AC in different ages by in vitro experimental study. Furthermore, dose-time-response of warfarin was positively correlated with AC score distribution and plasma levels of the SASP maker IL-6 among patients < 65 years, but not among patients ≥ 65 years. In addition, *in vitro* research suggested that warfarin treatment tended to deteriorate calcification in young VSMC at the early stage of calcification. Our results suggested that aging and warfarin-treatment were independently related to increased AC. Younger patients were more sensitive to warfarin-related AC than older patients, which was possibly due to accumulated warfarin-induced cellular senescence.

## 1. Introduction

Aortic calcification (AC) is a pathological condition with increasing prevalence of morbidity and mortality. AC is a process of osteoblast-like cell accumulation in the muscular layer of arteries. Enhanced stiffness of the arteries in AC might lead to severe vascular complications in the brain, heart, and kidneys. AC is a strong, independent predictor of cardiovascular disease (CVD) and cardiac adverse events. Risk factors of AC include advanced age, cigarette smoking, diabetes mellitus, hypertension, and kidney disease. In addition, cytokines and growth factors that also play a role in AC include proinflammatory cytokines (interleukin 6 (IL-6) and tumor necrosis factor-a (TNF-*α*)), osteoprotegerin, sclerostin, and matrix gamma carboxy glutamic acid-rich protein (MGP). Apart from the abovementioned traditional risk factors, warfarin, a vitamin K antagonist and currently the most frequently used anticoagulant in atrial fibrillation (AF) treatment, has been suggested to be associated with increased risk of AC [1]. Both in vitro and in vivo studies have demonstrated that warfarin could inactivate MGP, a vitamin K-dependent protein, and eventually lead to AC [2–5].

Aging is related to increased incidence, prevalence, and mortality associated with cardiovascular diseases. Accumulation of senescent cells within vascular tissues can potentially lead to aging-related biological dysfunction. Numerous studies have proved that aging is an independent risk factor of AC [6]. Besides, cellular senescence plays a pivotal role in the developmental pathophysiology of AC. It has been suggested that senescent vascular smooth muscle cells (VSMC) contribute to vascular dysfunction through induction to AC. However, the long-term effects of warfarin therapy and VSMC senescence have not been studied in detail.

It has been observed that there is a systemic increase level of secreted proteins with aging, which contain several proinflammatory cytokines, chemokines, tissue-damaging proteases, and growth factors. These secreted factors affect the microenvironment of tissue, in which they could propagate the stress response and regulate neighboring cells. These phenotypes are termed the senescence-associated secretory phenotype (SASP) [7]. The SASP is a critical intrinsic characteristic of cellular senescence resulting in a chronic low-grade state of inflammation that has been implicated in the development of several chronic diseases of aging including AC. Among SASP, cytokines and growth factors are important in the differentiation of senescent VSMC into calcified cells, and the effect of IL-6 on osteoblast differentiation and AC has been discussed previously [8]. So far, the long-term effects of warfarin treatment in AC and cellular senescence in different ages have not been studied in detail. However, recent studies showed that the association between warfarin treatment and AC might be influenced by the level of MGP which was not independently correlated with the mechanism of AC [4]. In this study, we conducted a retrospective analysis to investigate the contribution to warfarin use on AC and cellular senescence. Besides, we investigated the association between vitamin K and AC with data from the Multi-Ethnic Study of Atherosclerosis (MESA) cohort [6].

## 2. Materials and Methods

### 2.1. Study Population and Data Collection

The MESA is a multicenter, prospective study of risk factors affecting cardiovascular disease (CVD) progression. The 6655 MESA participants were enrolled from a multiethnical background, consisting of 6655 MESA participants that were enrolled from a multiethnical background, consisting of 2624 Caucasians, 1849 African Americans, 747 Chinese Americans, and 1435 Hispanics. The MESA recruited overlapping ethnic groups among field centers to minimize confounding by ethnicity by site. The design and study protocol of the MESA have been described elsewhere [9].

At the baseline examination, participants' dietary intake was assessed using a modified 120-item Block-style food-frequency questionnaire (FFQ) which includes Chinese and Hispanic foods to accommodate the MESA population. The FFQ inquired about serving size, frequency, and duration of intake for selected foods and beverages, allowing quantification of nutrient intake from supplements. Daily vitamin K intake from foods was estimated by multiplying the reported amount consumed by its nutrient content. Although FFQ cannot provide estimates of absolute vitamin K intake, it correctly ranked people within the population according to relative vitamin K intake. Adjustment for total dietary intake improves accuracy and reduces extraneous between-person variation in vitamin K intake, which might increase precision of the estimates due to cancellation of correlated measurement errors for total energy and food of interest.

This study cohort included 79 patients (40 males and 39 females) who attended Sun Yat-sen Memorial Hospital between January 2013 and November 2018. We used the following inclusion and exclusion criteria to determine the study cohort. The inclusion criteria included patients diagnosed with nonvalvular AF and were at risk for stroke (CHA2DS2-VASc score ≥ 2), aged from 18 to 74 years. Patients with a history of primary parathyroid disease, chronic kidney disease (CKD), heart failure, cirrhotic hepatic disease, acute or chronic inflammatory disease, malignancy, and diabetes mellitus (DM) were excluded from the study. Patients with hypertension were classified based upon clearly documented medical history with systolic blood pressure ≥ 140 mmHg or diastolic blood pressure ≥ 90 mmHg. Patients' baseline characteristics and the medication history of warfarin were extracted from the hospital information system. Data collection included demographic data, smoking history, medical history, and anthropometric measurements. The study protocol conformed to the ethical guidelines on the 1975 Declaration of Helsinki as reflected in a prior approval by the Ethics Committee of Sun Yat-sen University.

Dose-time-response models are used for the prediction of the response to conventional small drug molecules where the drug effects happen on much longer timescales, that is, months or years. In this study, dose-time-response of warfarin included two quantitative factors: dosage of warfarin (×1, mg) and postexposure time (×2, months). The dose-time-response value was calculated as ×1∗×2 (mg∗month).

### 2.2. Collection of Laboratory Parameters

All the patients were required for at least 10-hour fasting before the blood samples were drawn. Laboratory parameters which included measurements of serum calcium, phosphorus, alkaline phosphatase (ALP), total cholesterol (TC), triglyceride (TG), high-density lipoprotein cholesterol (HDL-c), low-density lipoprotein cholesterol (LDL-c), international normalized ratio (INR), fasting glucose, and high-sensitivity C-reactive protein (hsCRP) were analyzed using a standardized and certified TBA-120 autoanalyzer (Toshiba Medical Systems, Japan) in the institutional central laboratory of the Sun Yat-sen Memorial Hospital [10, 11].

### 2.3. Multidetector Row Computerized Tomographic Analysis

All the patients underwent a nonenhanced thoracoabdominal CT scan to evaluate AC. Calcification was identified as a plaque of ≥1 mm^2^ with a density of >130 Hounsfield units and quantified by the Agatston scoring method as described previously [12–14]. All imaging procedures were done on the same equipment using the same parameters. To measure AC, a single CT scan was performed using a scan collimation of 0.6 mm, slice thickness of 1 mm, reconstruction using 1 mm slice with 35 cm field of view, and normal kernel. Blood pressure and heart rate were monitored before the examination. Scans were read centrally by the SIEMENS Syngo CT Workplace, and calcification in the abdominal aorta was used for analysis. All CT scan data were analyzed at random order by two blinded and experienced investigators. According to the aortic calcium score, patients were grouped as having no detectable artery calcification (Agatston score = 0) and mild (1-100), moderate (101-400), and severe (>400) AC [5, 15].

### 2.4. Echocardiography

All patients underwent standard two-dimensional transthoracic echocardiography which was carried out on Doppler echocardiography (Vivid 3®, GE Vingmed Ultrasound; Haifa, Israel) using a 2.5 MHz transducer. Echocardiographic parameters included left ventricular ejection fraction, left atrium, and left ventricular posterior wall depth [16].

### 2.5. ELISA

Plasma samples from AF patients were collected for IL-6 laboratory testing. Peripheral blood samples were collected between 8 and 10 am using vials with ethylene diamine tetraacetic anticoagulant (EDTA) and stored at 4°C for a maximum of 2 h. Plasma was sampled after centrifugation at 3,000 rpm for 10 min at 4°C and stored at −80°C until analysis. Commercial enzyme-linked immunosorbent assay (ELISA) kits purchased from Meimian Biotechnology (Yancheng, Jiangsu, China) were used to measure human IL-6 level in human plasma samples according to the manufacturer's recommendations. Absorbances were measured in a Tecan Spark 10 M multimode microplate reader at 450 nm.

### 2.6. Cell Culture and Calcification Induction

VSMC were isolated from male Sprague–Dawley rats (3 weeks old, 40–60 g). Briefly, rats were euthanized using an intraperitoneal injection of sodium pentobarbital (60 mg/kg). The thoracic aorta was obtained, and the surrounding fat and connective tissues were discarded. It was slit longitudinally, and its lumen surface was scraped with a razor blade to remove the intima and then cut into 3–5 mm^2^ pieces. It was explanted lumen side down on collagen-coated culture dishes. After 7 days, tissue fragments were discarded and sprouted VSMC were collected (referred to as P0). Primary VSMC were cultured in Dulbecco's modified Eagle's medium (DMEM, Thermo Fisher Scientific) with 10% fetal bovine serum (FBS, Gibco) and 1% antibiotics (Gibco), at 37°C in 95% humidified air with 5% CO_2_. Calcification was induced by a solution to inorganic phosphate (Pi) (Na_2_HPO_4_ and NaH_2_PO_4_, pH 7.4, Sigma) added to serum supplemented-DMEM at concentrations of 3.0 mM (calcification medium).

### 2.7. Arterial Ring Calcification

Aortas (from the thoracic to the iliac arteries) were removed in a sterile manner from male Sprague–Dawley rats (150–200 g), and the adventitia and endothelium were carefully removed. The vessels were cut into 2–3 mm rings and placed in either calcification medium or normal culture medium at 37°C under 5% CO_2_ for 7 days, with medium changes once every 2 days. The arterial ring organ culture experiments were independently performed 3 times.

### 2.8. Alizarin Red S Staining

To observe calcium deposition, fixed VSMC or tissue sections were stained with 2% Alizarin Red S solution (Leagene) for 15 min at room temperature. Then, the samples were washed in PBS for 3 times. Samples were rinsed, and the stained calcium deposits were photographed.

### 2.9. Statistical Analysis

Data of the continuous variables were presented in mean and standard deviation (SD), skewed data in median and interquartile range, and categorical variables in absolute numbers and percentages. The baseline data between patients with AC and patients without AC were compared with the methods of the unpaired *t*-test, Mann-Whitney *U* test, chi-squared test, and Fisher's exact test for distributed variables. Spearman's rank correlation coefficient was used for exploring the correlation between the Agatston score and the baseline characteristics. Multivariable logistic regression analysis was used to explore the factors affecting calcification in patients with AF which was expressed in terms of odds ratio (OR) and 95% confidence interval (CI).

All statistical analyses were performed by using the software SPSS version 20.0 (SPSS, Inc., Chicago, Illinois, USA). And statistical significance was set to *P* < 0.05 [17, 18]. For all statistical tests, two-sided *P* = 0.05 was chosen to be the threshold of the statistical significance.

## 3. Results

### 3.1. Study Characteristics

At baseline, 51.8% of the population was male and the mean age was 61.90 years. Among the study population, 3356 (50.4%) participants had hypertension and 2058 (30.9%) had DM history. In addition, 3405 (51.1%) individuals were free from AC at baseline and AC was found in 3250 (48.9%) individuals.

6655 cases selected from the MESA were divided into two groups based on whether AC has been detected. The baseline characteristics across these 2 groups have been shown in Table 1. In the study population, participants with AC were older and had higher body mass index (BMI) and higher serum homocysteine and creatinine level than those without AC. Besides, participants with AC were more likely to be diabetic and had higher serum levels of proinflammatory markers and less accumulated vitamin K intake.

Similarly, baseline characteristics of the AF patients on warfarin are shown in Table 2. A total of 51.0% population was male, and the mean age was 64.0 years. Among the 79 patients, 40 (51.0%) participants had hypertension and 17 (21.0%) underwent statin therapy. In addition, 27 (34.2%) individuals were free from AC at baseline and AC was found in 52 (65.8%) individuals. The 79 patients were divided into the AC group and no-AC group. Participants with AC showed similar characteristics as the MESA cohort.

### 3.2. The Relationship among Vitamin K Intake, AC, and SASP

**Figure 1:**
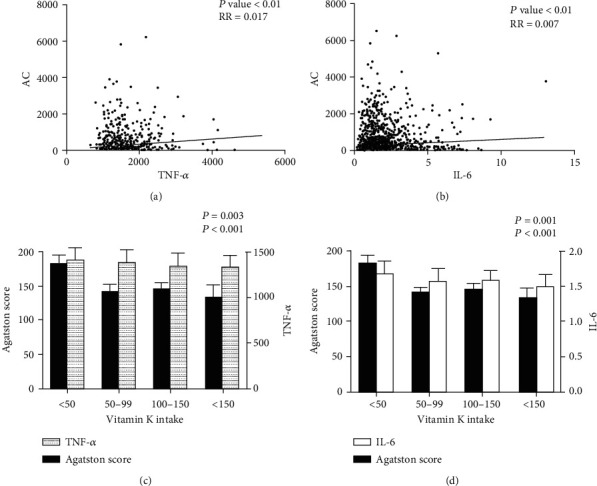
Correlation between AC score and SASP in the MESA. Each dot referred to one patient. (a) Linear regression analysis between AC score and TNF-*α*. (b) Linear regression analysis between AC score and IL-6. (c, d) Comparison of AC score, IL-6, and TNF-*α* between different vitamin K intake groups.

To establish the relationship among warfarin, senescence, and AC, 6655 cases from the MESA were analyzed. Since dose information of warfarin was not available in the MESA, accumulated vitamin K intake was analyzed to mimic the effect of warfarin. As shown in Figures 1(a) and 1(b), SASP marker IL-6 (*P* < 0.01, RR = 0.007) and TNF-*α* (*P* < 0.01, RR = 0.017) were both positively correlated with AC (Figures 1(a)–1(b)) according to a linear regression analysis. Further, we divided participants into 4 groups depending on vitamin K intake. Statistical results showed that intake of vitamin K was negatively related to Agatston score (*P* < 0.01). Interestingly, individuals with higher vitamin K intake were more likely to show lower IL-6 (*P* < 0.01) and TNF-*α* (*P* < 0.01) level in the circulation (Figures 1(c) and 1(d)).

### 3.3. Comparison of Demographic and Clinical Characteristics between Absence and Presence of AC in AF Patients

Clinical data from 79 patients with NVAF were obtained for this analysis. These patients were divided as the noncalcification group (Agatston = 0) and calcification group (Agatston > 0) depending on the Agatston score of AC. Comparisons between demographic, biochemical parameters, and medication history across these two groups are shown in Table 2. The mean age of the calcification group was significantly higher than that of the noncalcification group (67 vs. 59 years, *P* = 0.001). The calcification group also tended to have significantly higher levels of ALP and hsCRP in comparison to the noncalcification group (ALP, 77 U/L (57, 93) versus 62 U/L (47, 74), *P* = 0.001; hsCRP, 5.26 mg/dL (1.67, 17.91) versus 1.49 mg/dL (0.88, 3.32), *P* = 0.005, Table 2). Interestingly, the dose and the duration of the warfarin treatment and INR control level showed no significant differences between the two groups (*P* > 0.05, Table 2). Moreover, no statistical significance was found in BMI, smoking, systolic blood pressure (SBP) and diastolic blood pressure (DBP), total cholesterol (TC), LDL-c, HDL-c, triglycerides, fasting glucose, and serum calcium and phosphorus (*P* > 0.05, Table 2).

### 3.4. Correlation between AC and Clinical, Biochemical, and Echocardiographic Parameters and Medication History in AF Patients

To better understand the relationship between clinical variables and AC in AF patients, correlation analyses were performed. The results are shown in Table 3. It was found that age, ALP, and hsCRP were positively correlated with AC (age, *r* = 0.464, *P* < 0.001; ALP, *r* = 0.253, *P* = 0.025; hsCRP, *r* = 0.322, *P* = 0.004). However, no significant correlation was observed among AC and the dose of warfarin treatment, the duration of warfarin treatment, and the value of INR (dose of warfarin, *r* = −0.020, *P* = 0.862; duration of warfarin, *r* = −0.021, *P* = 0.852; and INR, *r* = −0.056, *P* = 0.624, Table 3).

### 3.5. Risk Factor Analysis of AC in AF Patients

To elucidate the independent predictors of AC in AF patients, multivariable logistic regression analysis was performed. As expected, age and ALP were independent risk factors of AC (OR = 1.125, *P* = 0.008, Table 4). Of note, neither the dose nor the duration of the warfarin treatment was shown to be an independent risk factor of AC in AF patients (dose of warfarin, *P* = 0.563; duration of warfarin, *P* = 0.462, Table 4).

### 3.6. Correlation between Warfarin Use and AC in Different Age Groups of AF Patients

**Figure 2:**
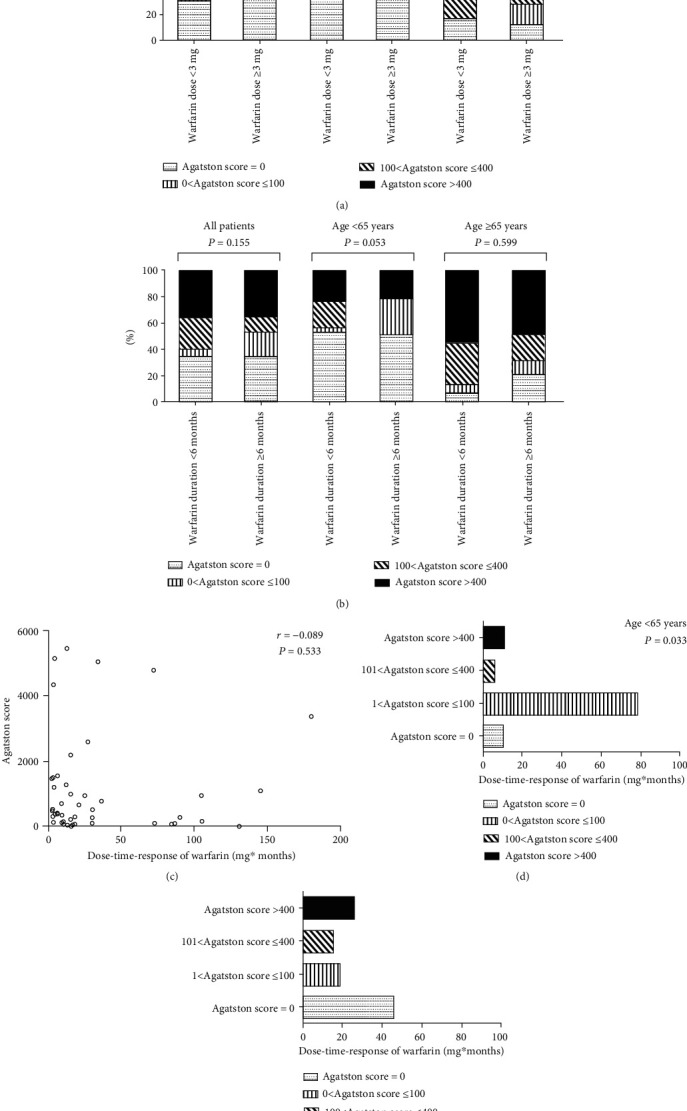
(a, b) Abdominal AC score categories in patients with different doses and durations of warfarin according to age. Comparison between the low-dose group (warfarin dose < 3 mg) and high-dose group (warfarin dose ≥ 3 mg) in different age groups (a). Comparison between the short-duration group (warfarin duration < 6 months) and long-duration group (warfarin duration ≥ 6 months) in different age groups (b). (c) Correlation between dose-time-response of warfarin and AC score in atrial fibrillation patients. Each circle referred to one patient. (d, e) AC score categories in patients with different doses and durations of warfarin according to age. Comparison between different aortic calcium score groups in patients with age < 65 years (d). Comparison between different aortic calcium score groups in patients with age ≥ 65 years (e).

To exclude the influence of age on the association between use of warfarin and AC, participants were divided into 2 groups. We classified the degree of calcification according to the Agatston score. The results showed that within the two age groups, no significant differences of AC were found in doses and durations of warfarin treatment (Figures 2(a) and 2(b)). To further explain the relationship between warfarin treatment and AC in AF patients, the dose-time-response of warfarin was introduced. Dose-time-response of warfarin was defined as the product of dose and duration of warfarin. As shown in Figure 2(c), there was no significant correlation between dose-time-response of warfarin and the Agatston score in all AF patients (*r* = −0.089, *P* = 0.533). After dividing the patients into two age groups and ranged calcification groups according to Agatston score, there was still no significant in patients ≥ 65 years (*P* = 0.885, Figure 2(e)). However, the dose-time-response of warfarin was significantly associated with increased Agatston score in the mild calcification group (Agatston score = 1-100) in patients < 65 years (*P* = 0.033, Figure 2(d)), which suggested that accumulating use of warfarin may promote low-grade calcification in young patients.

### 3.7. Correlation between Dose-Time-Response of Warfarin and SASP Marker Expression in Different Ages of AF Patients

**Figure 3:**
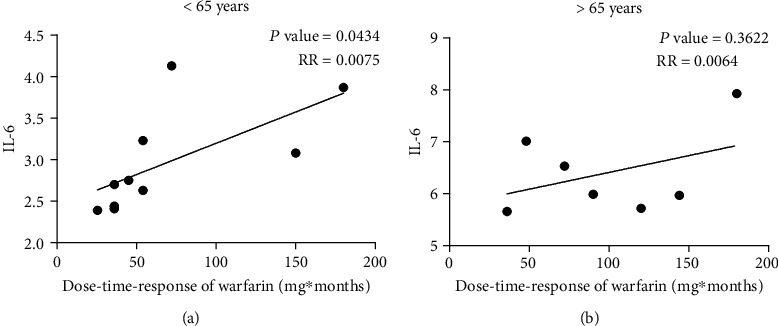
The correlation between IL-6 and dose-time-response of warfarin. IL-6 release in plasma was measured by ELISA. Results are expressed as *μ*g/L. (a) Correlation between dose-time-response of warfarin and IL-6 level in patients with age < 65 years. (b) Correlation between dose-time-response of warfarin and IL-6 level in patients with age ≥ 65 years.

The proinflammatory cytokine IL-6 is a proven marker of SASP. In order to explore the relationship between warfarin treatment and IL-6 concentration, other 17 plasma samples from AF patients were collected. IL-6 level was detected by an ELISA kit. As shown in Figure 3, the dose-time-response of warfarin was positively correlated with plasma IL-6 level in patients < 65 years but not in patients ≥ 65 years. The correlation coefficient was 0.0075 and 0.0055, respectively. It is noteworthy that the basal level of IL-6 was much higher in patients ≥ 65 years. These data suggested that warfarin-associated cell senescence was more sensitive in younger patients.

### 3.8. Warfarin Enhanced the Susceptibility to Pi-Induced Calcification

**Figure 4:**
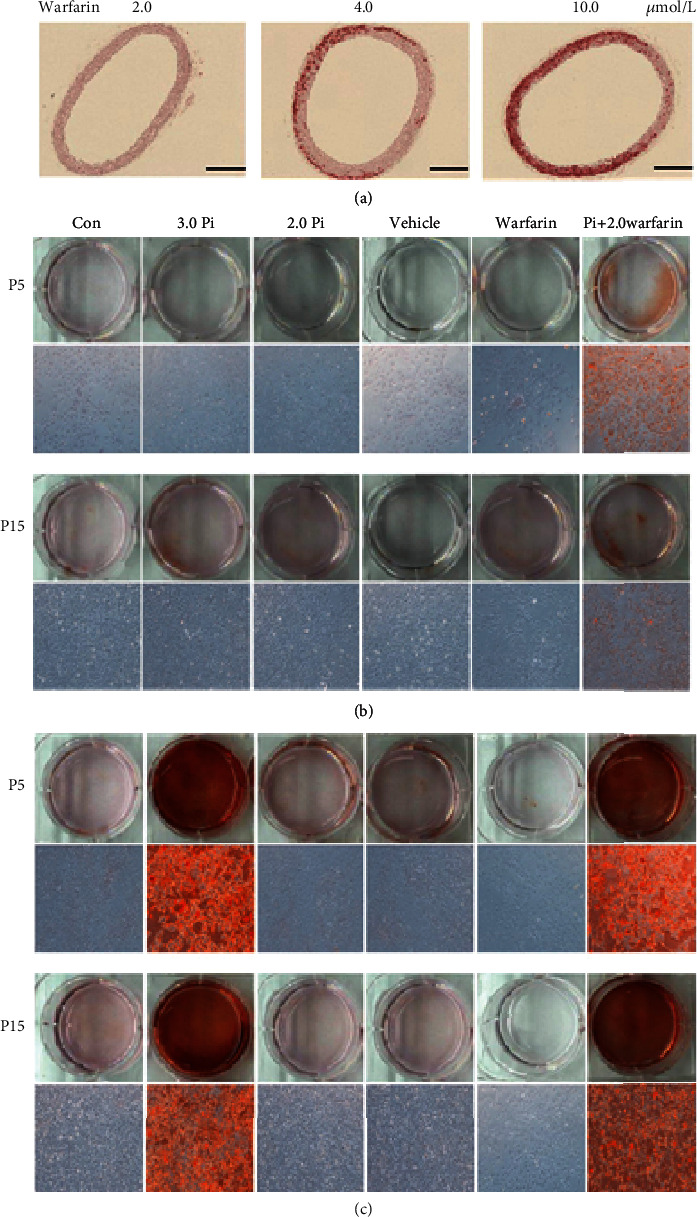
Warfarin enhanced the susceptibility of Pi-induced calcification. (a) Effect of warfarin on Pi-induced AC in cultured explants of aorta. Pieces of rat aorta were cultured in calcification medium for 7 days. The calcified lesions were examined by von Kossa and Alizarin Red S staining. Scale bar: 100 *μ*m. (b, c) Passage 5 (P5) and passage 15 (P15) VSMC were treated with phosphate (2.0 mmol/L), vehicle (methanol), and warfarin (2.0 *μ*mol). Representative Alizarin Red staining for VSMC. Calcification in day 3 (b) and day 7 (c). Treatment with warfarin enhanced the VSMC calcification in both the senescent and young VSMC. Of note, calcification was further deteriorated by the addition of warfarin in the young VSMC compared with vehicle control in day 3.

To investigate whether warfarin promotes Pi-stimulation-induced vascular calcification ex vivo, rat aorta rings were cultured with 0.65 mM Pi and different concentrations of warfarin for 7 days. Under normal phosphate and low-grade warfarin conditions, no calcification was observed in cultured aortas, and vascular smooth muscle layers of the aorta showed calcium deposition in the presence of 4.0 *μ*M or higher concentration of warfarin (Figure 4(a)). We used VSMC in early passage (passage 5 (P5)) to mimic young cells, and continuously passage cultured VSMC were used as the replicative senescent VSMC. In both young and senescent VSMC, incubation in calcification medium with a higher concentration of Pi (3.0 mM) resulted in calcium deposition within 7 days but not in a relatively low concentration (2.0 mM). However, calcification occurred when VSMC were cultured in the medium containing 2.0 mM high phosphate and 2.0 *μ*M warfarin. Warfarin-induced calcification was not different between the senescent and the control young VSMC in day 7. Interestingly, young VSMC showed increased calcification in day 3, which suggested that young VSMC were more sensitive to warfarin-induced calcification in the early stage (Figures 4(b) and 4(c)).

## 4. Discussion

The present study demonstrated that the association of warfarin use with AC differs in different age groups of patients. Specifically, warfarin adversely affects younger (<65 years) patients more than older (≥65 years) patients, and this is possibly due to the fact that warfarin-associated senescence was more sensitive in younger patients. In vitro study also revealed that young VSMC are more sensitive to warfarin-induced low-grade calcification.

In this paper, we found that SASP was positively related to AC from the MESA cohort, and initially suggested that SASP level was inversely correlated with vitamin K intake. Vitamin K has been widely considered an inhibitory factor in the progression of AC. There is accumulating data suggesting the relationship between vitamin K status and AC, including observational studies and the MESA cohort, which supports that our conclusion is solid. For example, Geleijnse et al. showed that dietary intake of vitamin K was inversely associated with AC and all-cause mortality in a cohort of 4807 [19]. In the MESA, we discovered that the accumulated intake of vitamin K was negatively related to AC, which demonstrated that warfarin may aggravate AC by downregulating the level of vitamin K in plasma. In addition, IL-6 and TNF-*α* levels were positively correlated with AC. When we divided individuals depending on vitamin K intake and analyzed the relationship among vitamin K, AC, and SASP, results showed that Agatston score and SASP level decreased with the increase of vitamin K intake. It suggested that warfarin may participate in SASP regulation during AC process. In further subgroup analysis, we demonstrated that age but not warfarin use was independently related to AC in patients with AF. Furthermore, we divided the AF patients into two different age groups (over or less than 65) and noted that the dose-time-response of warfarin was positively correlated with AC score and IL-6 level in younger AF patients. Interestingly, Weijs et al. reported that the intake of vitamin K antagonists would increase the levels of coronary artery calcification (CAC) [5]. Similar findings were demonstrated in CKD and diabetic patients [20–23]. However, our results demonstrated that warfarin treatment did not aggravate AC in AF patients. Compared to these studies, there are several distinctions in our study. One possibility is that, in our study, all the AF patients were at low risk without other underlying diseases. However, the participants from other studies were diagnosed with other AC-associated comorbidities such as CKD and DM [22, 24, 25]. With the disorder of the homeostasis, such as the vitamin D deficiency and hyperphosphatemia, AF patients might aggravate AC without the use of warfarin [26]. Besides, previous studies mainly concentrated on CAC in AF patients [27, 28] instead of AC in this article. It has been highlighted that AC preceded the occurrence of CAC in patients on hemodialysis and systemic chronic inflammation had more deteriorative effect on the AC than CAC [29, 30]. Furthermore, the present observation was a single-center study, and all of the AF patients in our study were Asians, while in most of the previous studies, the participants were Europeans [31, 32, 33]. According to the guidelines of the management of AF in 2010 European Society of Cardiology, the level of INR should be controlled from 2.0 to 3.0. However, in China, most of the AF patients have their INR controlled in the range of 1.6 to 2.5, which is much lower than the guideline suggested [25]. In our study, the average level of the INR was 1.39. Besides, anticoagulant responses to warfarin vary among patients, based on their genotype. Gaikwad et al. demonstrated that the genotype of CYP2C9 and VKORC1 in East Asian countries was different from that seen among Europeans [30]. These two genes play an important role in warfarin dose variation and its related adverse effects. Since the reactivity, the intake, and the drug metabolism of warfarin in East Asians are much different from those in Europeans, the effects of warfarin on AC might be different in these two areas.

Our data from Chinese AF patients and the MESA showed that aging is an independent risk factor of AC. Early vascular aging, which particularly focuses on vascular inflammation, stem cells, and calcification, has been of interest [34, 35]. Cellular senescence is a program of cell cycle which is initiated in response to a variety of stimuli, such as oncogene activation (oncogene-induced senescence (OIS)) or replicative exhaustion (replicative senescence (RS)) [36]. SASP was first revealed by Vijg and Campisi and their group in 2008 [37]. They demonstrated that senescent cells secreted a myriad of factors associated with inflammation and oncogenesis. Senescent cells are characterized by a proinflammatory, oxidant SASP that occurs within a few days of senescence. Classical SASP inflammatory mediators include IL-6, IL-8, TNF-*α*, and monocyte chemotactic protein 1 (MCP-1). According to MESA data, IL-6 and TNF-*α* level was positively related to AC. The proinflammatory cytokine IL-6 is upregulated in the SASP of cells and plays a causal role in disease pathology. Kuilman et al. demonstrated that IL-6 has a crucial role in the generation of OIS since its depletion abolished oncogene-induced cellular senescence. Our statistical result (Figures 1(c) and 1(d)) revealed that IL-6 showed more relevance to vitamin K intake than TNF-*α*. Thus, we investigated the relationship between IL-6 level and warfarin intake. Interestingly, we found that warfarin-treatment influenced plasma IL-6 concentration in AF patients, especially in the younger (age ≤ 65years). It is suggested that within the vascular aging, senescent cells have been associated with warfarin treatment. Therefore, warfarin may induce vascular cell senescence and likely contributes to the spread of vascular inflammation and oxidative stress via SASP. Thus, warfarin is likely to induce chronic low-grade inflammation in nonsenescent VSMC and then enhance vascular aging and calcification.

Treatment with warfarin enhanced calcification; compared with senescent VSMC, it worked sensitively in young VSMC at the early stage. Previous studies have reported that treatment with warfarin deteriorated VSMC calcification only in the young VSMC, not in the senescent VSMC [38], which is consistent with our finding. This different effect of warfarin is probably due to the loss of MGP in the senescent VSMC.

There are several limitations that should be highlighted in this study. This study is a small case-control study so a cause-and-effect relationship could not be concluded; more large-scale randomized clinical trials and basic researches are needed to corroborate the current observations. Besides, the genotype of the patients might be involved in the interaction between warfarin and AC. Further long-term large-scale randomized study is needed to corroborate the current observations.

## 5. Conclusions

Warfarin impacts AC more in younger patients (<65 years) than in older patients (≥65 years), and this is possibly due to the increased senescence by warfarin in this population. These findings suggest that warfarin use should be considered more cautiously in younger AF patients.

## Figures and Tables

**Table 1:** Baseline characteristics according to absence and presence of AC in patients from MESA.

	AC, *N* = 3250	No AC, *N* = 3405	*P* value
Demographics			
Age (yr)	66 ± 9	58 ± 9	<0.01
Gender, male (%)	59	45	0.03
Metabolic syndrome (%)	40	38	0.04
Hypertension (%)	53	48	0.02
Diabetes mellitus (%)	34	28	0.02
Body mass index (kg/m^2^)	28 ± 5	28 ± 6	0.17
SASP			
IL-6 (mg/dL)	1.6 ± 1	1.3 ± 1	<0.01
TNF-*α* (mg/dL)	1469 ± 461	1294 ± 383	<0.01
Biochemical index			
Total homocysteine (*μ*mol/L)	9.9 ± 3.6	8.5 ± 3.5	<0.01
Glucose (mmol/L)	101 ± 33	99 ± 32	0.03
Cholesterol (mmol/L)	195 ± 35	193 ± 34	0.35
Serum creatinine (*μ*mol/L)	1.0 ± 0.2	0.9 ± 0.5	0.04
Vitamin K intake (*μ*g)	126.91 ± 130.55	135.86 ± 125.13	<0.01
Calcification score			
Agatston score	273 ± 53.2	0	<0.01

Data are presented as mean ± SD. *P* values are from independent-sample *t*-test, Mann-Whitney *U* test, chi-squared test, and Fisher's exact test for appropriate data between group AC and group no AC.

**Table 2:** Baseline characteristics according to absence and presence of AC in AF patients with warfarin treatment.

Characteristics	Overall (*n* = 79)	AC (*n* = 52)	No AC (*n* = 27)	*P* value
Demographics				
Male (%)	40 (51)	26 (50)	14 (52)	0.877
Age (years)	64 ± 10.1	67 ± 8.8	59 ± 10.5	0.001^∗^
BMI (kg/m^2^)	24.8 ± 3.6	25 ± 3.9	24 ± 2.8	0.502
Smoking (%)	18 (23)	11 (21)	7 (26)	0.634
SBP (mmHg)	126 (116, 140)	129.5 (120, 140)	120 (108, 142)	0.197
DBP (mmHg)	77 (68, 84)	76 (67, 85)	78 (71, 84)	0.290
HR (bpm)	80 (70, 88)	80 (72, 89)	80 (70, 88)	0.709
AF (%)	79 (100)	52 (100)	27 (100)	1
Hypertension (%)	40 (51)	29 (56)	11 (41)	0.208
CHD (%)	18 (23)	13 (25)	5 (19)	0.517
Biochemical index				
ALP (U/L)	73 (54, 82)	77 (57, 93)	62 (47, 74)	0.001^∗^
Ca (mmol/L)	2.22 ± 0.11	2.21 ± 0.12	2.23 ± 0.10	0.604
P (mmol/L)	1.12 ± 0.20	1.14 ± 0.23	1.10 ± 0.15	0.433
GLU (*μ*mol/L)	5.2 ± 1.5	5.4 ± 1.8	4.9 ± 0.6	0.583
TC (mmol/L)	4.57 ± 1.2	4.47 ± 1.17	4.77 ± 1.4	0.320
TG (mmol/L)	1.62 ± 1.1	1.49 ± 1.01	1.87 ± 1.3	0.141
HDL-c (mmol/L)	1.15 ± 0.31	1.16 ± 0.31	1.15 ± 0.32	0.996
LDL-c (mmol/L)	2.78 ± 0.85	2.71 ± 0.83	2.94 ± 0.87	0.251
hsCRP (mg/L)	3.02 (1.34, 12.76)	5.26 (1.67, 17.91)	1.49 (0.88, 3.32)	0.005^∗^
INR	1.39 ± 0.49	1.35 ± 0.47	1.47 ± 0.54	0.357
Echocardiography				
LA (mm)	40 ± 6.0	41 ± 6.4	39 ± 5.1	0.121
LVPWT (mm)	10 ± 1.3	10 ± 1.4	9 ± 0.9	0.171
LVEF (%)	64 ± 10.4	65 ± 9.5	63 ± 11.9	0.272
Medication history				
Warfarin dose (mg)	2.8 ± 0.9	2.8 ± 1.0	2.8 ± 0.6	0.878
Warfarin duration (m)	5 (1, 24)	5 (2, 27)	5 (1, 24)	0.921
Warfarin (%)	79 (100)	52 (100)	27 (100)	1
Clopidogrel (%)	8 (10)	5 (10)	3 (11)	0.835
Statin (%)	17 (21)	13 (25)	4 (15)	0.299

Data are presented as mean ± SD. Male, smoking, hypertension, clopidogrel, statin, and CHD are presented as *n* (%). SBP, DBP, HR, ALP, hsCRP, and warfarin duration are presented as median (interquartile range). *P* values are from independent-sample *t*-test, Mann-Whitney *U* test, chi-squared test, and Fisher's exact test for appropriate data between group AC and group no AC (^∗^*P* < 0.05). AF: atrial fibrillation; ALP: alkaline phosphatase; BMI: body mass index; DBP: diastolic blood pressure; GLU: glucose; HCRP: high-sensitivity C-reactive protein; HDL-c: high-density lipoprotein cholesterol; HR: heart rate; INR: international normalized ratio; LA: left atrium; LDL-c: low-density lipoprotein cholesterol; LVEF: left ventricular ejection fraction; LVPWT: left ventricular posterior wall thickness; SBP: systolic blood pressure; TC: total cholesterol; TG: triglyceride.

**Table 3:** Correlation analysis of aortic Agatston score in AF patients.

Characteristics	Aortic Agatston score	
	*r*	*P*
Age (years)	0.464	<0.001^∗^
BMI (kg/m^2^)	-0.75	0.512
SBP (mmHg)	0.84	0.460
DBP (mmHg)	-2.07	0.670
Smoking (%)	-0.28	0.804
Ca (mmol/L)	-0.141	0.216
P (mmol/L)	0.059	0.608
ALP (U/L)	0.253	0.025^∗^
GLU (umol/L)	0.114	0.318
HDL-c (mmol/L)	-0.119	0.295
LDL-c (mmol/L)	-0.250	0.026^∗^
hsCRP (mg/L)	0.322	0.004^∗^
Warfarin dose (mg)	-0.020	0.862
Warfarin duration (month)	-0.021	0.852
INR	-0.056	0.624

*r* value for spearman correlation coefficients (^∗^*P* < 0.05). ALP: alkaline phosphatase; BMI: body mass index; DBP: diastolic blood pressure; GLU: glucose; hsCRP: high-sensitivity C-reactive protein; HDL-c: high-density lipoprotein cholesterol; INR: international normalized ratio; LDL-c: low-density lipoprotein cholesterol; SBP: systolic blood pressure.

**Table 4:** Multivariable logistic regression analysis for the independent risk factor of AC in AF patients using warfarin.

Characteristics	OR	95% CI	*P* value
Age	1.125	1.032-1.227	0.008^∗^
BMI	1.170	0.971-1.409	0.099
Smoking (%)	1.218	0.301-4.925	0.782
ALP (U/L)	1.037	1.004-1.071	0.027
GLU (*μ*mol/L)	1.609	0.909-2.847	0.103
LDLC (mmol/L)	0.773	0.377-1.583	0.481
hsCRP (mg/L)	0.995	0.967-1.024	0.736
Warfarin (mg)	1.239	0.599-2.561	0.563
Warfarin (month)	0.985	0.945-1.026	0.462
INR	0.817	0.269-2.484	0.721

OR value for multivariable logistic regression (^∗^*P* < 0.05). ALP: alkaline phosphatase; BMI: body mass index; GLU: glucose; hsCRP: high-sensitivity C-reactive protein; INR: international normalized ratio; LDL-c: low-density lipoprotein cholesterol.

## Data Availability

All data generated or analyzed during this study are included in the article. The original clinical and experimental data used to support the findings of this study are available from the corresponding author upon request.
